# Regulation of the integrin αVβ3- actin filaments axis in early osteogenesis of human fibroblasts under cyclic tensile stress

**DOI:** 10.1186/s13287-021-02597-y

**Published:** 2021-10-07

**Authors:** Yan Peng, Rongmei Qu, Yanting Feng, Xiaolan Huang, Yuchao Yang, Tingyu Fan, Bing Sun, Asmat Ullah Khan, Shutong Wu, Jingxing Dai, Jun Ouyang

**Affiliations:** 1 Guangdong Provincial Key Laboratory of Medical Biomechanics and Department of Anatomy, School of Basic Medical Science, Southern Medical University, Guangzhou, 510000 China; 2 Department of Ophthalmology, The First Affiliated Hospital of Guangzhou Medical University, Guangzhou, 510000 Guangdong China

**Keywords:** Osteogenesis, Integrin αVβ3, Tensile stress, Cellular mechanotransduction, Fibroblast

## Abstract

**Background:**

Integrins play a prominent role in osteogenic differentiation by transmitting both mechanical and chemical signals. Integrin expression is closely associated with tensile stress, which has a positive effect on osteogenic differentiation. We investigated the relationship between integrin αVβ3 and tensile stress.

**Methods:**

Human fibroblasts were treated with c (RGDyk) and lentivirus transduction to inhibit function of integrin αVβ3. Y-15, cytochalasin D and verteporfin were used to inhibit phosphorylation of FAK, polymerization of microfilament and function of nuclear YAP, respectively. Fibroblasts were exposed to a cyclic tensile stress of 10% at 0.5 Hz, once a day for 2 h each application. Fibroblasts were harvested on day 4 and 7 post-treatment. The expression of ALP, RUNX2, integrin αVβ3, β-actin, talin-1, FAK, vinculin, and nuclear YAP was detected by Western blot or qRT-PCR. The expression and distribution of integrin αVβ3, vinculin, microfilament and nuclear YAP.

**Results:**

Cyclic tensile stress was found to promote expression of ALP and RUNX2. Inhibition of integrin αVβ3 activation downregulated the rearrangement of microfilament and the expression of ALP, RUNX2 and nuclear YAP. When the polymerization of microfilament was inhibited the expression of ALP, RUNX2 and nuclear YAP were decreased. The phosphorylation of FAK induced by cyclic tensile stress reduced by the inhibition of integrin αVβ3. The expression of ALP and RUNX2 was decreased by inhibition of phosphorylation of FAK and inhibition of nuclear YAP.

**Conclusions:**

Cyclic tensile stress promotes osteogenesis of human fibroblasts via integrin αVβ3-microfilament axis. Phosphorylation of FAK and nuclear YAP participates in this process.

**Supplementary Information:**

The online version contains supplementary material available at 10.1186/s13287-021-02597-y.

## Introduction

Large bone defects (with a fracture space > 2.5 cm), whether caused by trauma, tumors, or infection, require external bone repair. Bone grafting is the currently favored approach to repairing large bone defects, but it has several drawbacks, including limited source material and donor site morbidity [1–3]. An alternative approach is bone tissue engineering, which uses biomaterials and stem cells that can be easily propagated and induced to differentiate into osteocytes that contribute to new bone formation [4, 5].

Fibroblasts are a common adherent cell type within the mesenchymal stroma [6]. Fibroblasts and mesenchymal stem cells have similar MSC markers and can differentiate into cells of osteogenic, adipogenic, and chondrogenic lineages [7, 8]. In fibrodysplasia ossificans progressiva model, Micha D et al. have observed that fibroblasts from patients show an enhanced potential for osteogenic differentiation in agreement with the heterotopic ossification characterizing this disease [9]. In another approach, the subcutaneous ectopic bone formation in mice after implantation of the epigenetically modified fibroblasts [10]. Further, differentiation of fibroblasts toward the osteoblast lineage was also demonstrated with treatment of osteoclast-conditioned media, and TGF-β was shown to improve the capacity of fibroblasts for osteogenic differentiation [11, 12]. The similarities of fibroblasts and mesenchymal stem cells support the use of fibroblasts as an appropriate cell type to study osteogenesis [13]. When stem cells are in a microenvironment composed of biomaterials, growth factors and tissues, the appropriate mechanical stimuli can promote osteogenic differentiation of stem cells [14]. Clinical and basic studies have shown that external mechanical stimulation, such as increased tension in response to stretch, can affect osteogenic differentiation of stem cells [15, 16]. Mechanotransduction is the process by which mechanical stimuli are converted into biochemical signals, often via integrins, which then affect downstream cellular activities [17].

Integrins are transmembrane receptors that exist on the cell surface and are functional heterodimers composed of *α* and *β* subunits linked by non-covalent bonds [18, 19]. Integrins mediate connections between cells and the extracellular matrix, from which mechanical stimulation can be transduced into the cell via integrins, which then initiate and regulate intracellular osteogenic signaling pathways [20, 21]. Integrins bind to extracellular matrix ligands via their large extracellular domains and to the cytoskeleton via short unstructured cytoplasmic tails capable of interacting with multiple intracellular proteins. Aggregation and activation of integrins recruit and activate intracellular signaling proteins such as focal adhesion kinase (FAK), vinculin and talin, which contribute to focal adhesions and influence differentiation of stem cells [22–24].

Yes-associated protein (YAP) is a transcriptional coactivator that promotes cell proliferation and regulates differentiation of stem cells by binding to TEAD (TEA domain DNA-binding family of transcription factors) DNA-binding proteins that can control transcription [25–27]. Studies have shown that manipulation of F-actin levels through knockdown of regulators of the actin cytoskeleton or treatment with F-actin-inhibitory drugs significantly affects YAP activity [28]. However, how mechanical stimulation influences YAP through the integrin-microfilament axis to regulate stem cell osteogenic differentiation remains unclear.

We investigated the relationship between integrin αVβ3 and early stage of osteogenic differentiation induced by cyclic tensile stress (CTS). Activation of integrin αVβ3 leads to rearrangement of actin filaments and regulated the expression of nuclear YAP. We also found that FAK participated in the formation of focal adhesions, and phosphorylation of FAK mediated by integrin αVβ3 played a role in early stage of osteogenic differentiation induced by CTS. This study shows that integrin αVβ3 may serve as a therapeutic target for bone repair.

## Materials and methods

### Chemicals and reagents

c(RGDyk) (10 μM, dissolve in medium) was purchased from Selleck (Shanghai, China). RGD (10 μM, dissolve in medium) and RGE (10 μM, dissolve in medium) peptides were purchased from A-Peptide (Shanghai, China). Y-15(2 μM, dissolve in medium), cytochalasin D (Cyto D, 0.2 μg/mL, dissolve in DMSO) and verteporfin (VP, 5 μM, dissolve in medium) were purchased from MedChemExpress (NJ, the USA).

### Cell culture

Human skin fibroblasts were purchased from ScienCell research laboratories (San Diego, CA, the USA, Cat #2320) and isolated from adult human skin. Human skin fibroblasts were cultured in DMEM (Gibco, Grand Island, NY, the USA) at 37 °C in a 5% CO^2^ atmosphere. The medium was supplemented with 10% heat‐inactivated fetal bovine serum (Gibco) and 1% mixture of penicillin and streptomycin (Gibco). The 11th generation of fibroblasts will be used in subsequent experiments.

After 48 h of cell passage, all inhibitors were applied for 24 h, followed by tensile stress. During stretching, the medium which include inhibitors should be changed every 2 days to maintain the working concentration.

### Tensile stress loading

Cells were plated at a density of 1 × 10^5^ cells per dish on collagen I-coated silicone membrane plates (Bioflex, Flexcell International, NC, the USA) and cultured for 2 days before beginning experiments. Cyclic tensile stress was applied to fibroblasts plated on six-well Bioflex Collagen I-coated plates using the Flexcell FX-5000 system (Flexcell International). A regimen of 10% tensile strain was delivered at 0.5 Hz for 2 h each day. Cells used for CCK8 and ALP (alkaline phosphatase) staining, Western blotting, qRT-PCR and immunofluorescence were processed immediately after application of cyclic tensile stress. Cells cultured under similar conditions but without cyclic stretch were used as unstretched controls.

### ALP staining

Cells were washed twice with phosphate-buffered saline (PBS) and fixed for 15 min with 4% paraformaldehyde. Thereafter, the cells were rinsed twice with PBS and added to an ALP reaction solution (Beyotime, Shanghai, China). After incubation for 30 min at 37 °C, the reaction solution was removed, and the cells were rinsed twice with PBS. The stained cells were observed under optical microscope (OLYMPUS, Japan).

### CCK8 experiment

Cells were rinsed twice with PBS and added to a CCK8 reaction solution (Beyotime). After incubation for 1 h at 37 °C, the absorbance of reaction solution was detected by microplate reader.

### Lentivirus transduction

Green fluorescent protein-labeled plasmid vectors (negative control (NC)) (Cat #CON077), hsa-ITG-AV (integrin αV) (Cat #51830) and hsa-ITG-B3 (integrin β3) (Cat #16375) were synthesized and packaged into lentivirus by Shanghai GeneChem Co. Ltd. Cell suspensions were prepared at a density of 1 × 10^4^ cells/mL growth medium (GM), and 2 mL was seeded into a 6-well plate. After 24 h, when the cell density reached 30–40%, GM supplemented with 40 μL/mL HistransG P and lentivirus (multiplicity of infection 40) was added to each well. After 12 h, the medium was changed, and the cells were incubated for 3 days. The extent of transfection was observed under an inverted phase-contrast fluorescence microscope on the fifth day. To screen successfully transfected cells, the medium was changed to GM containing 3 μg/mL puromycin for 3 days. After 3 days of screening with puromycin, fibroblasts were replaced with growth medium and cultured normally.

### RNA extraction and quantitative real-time PCR

Total cellular RNA was extracted using Trizol lysis buffer. RNA was reverse transcribed to cDNA using the Qiagen (Shanghai, China) miRNA reverse transcription (miScript II RT Kit) and thermo reverse transcription kits. Expression of integrin αV, integrin β3, ALP, RUNX2, Talin-1, FAK, vinculin, β-actin and GAPDH was detected by quantitative real-time PCR (qRT-PCR) by using cDNA as a template. The qRT-PCR was performed using an ABI 7500 qRT-PCR system (Applied Biosystems). The specific primer sequences used are shown in Table 1.

**Table 1:** Primers used in the qRT-PCR

Gene	Forward primer	Reverse primer
GAPDH	TCGGAGTCAACGGATTTGGT	TTCCCGTTCTCAGCCTTGAC
RUNX2	GAGATCATCGCCGACCAC	TACCTCTCCGAGGGCTACC
ALP	ACCATTCCCACGTCTTCACATTTG	AGACATTCTCTCGTTCACCGCC
ITG AV	CCGAAGCTCAGCCCTCTTG	GAAAAGCCATCGCCGAAGTG
ITG B3	ACCAGTAACCTGCGGATTGG	CTCATTGAAGCGGGTCACCT
Talin-1	GGAAAAGTTGCGGGGCATAG	CAAGAACACAGGCCGTTTGG
FAK	CAGGGTCCGATTGGAAACCA	CTGAAGCTTGACACCCTCGT
Vinculin	CGCTGAGGTGGGTATAGGTG	GTAGCTTCCCGATGCAAGGA

### Western blot analysis

Cells were harvested and washed twice with PBS. Cellular protein was extracted using radio-immunoprecipitation assay lysis buffer. Protein was separated by 10% SDS polyacrylamide gel electrophoresis and transferred onto polyvinylidene difluoride (PVDF) membranes. The PVDF membranes were removed and blocked with 5% (w/v) skimmed milk powder for 1 h after electrophoresis was complete. All membranes were incubated at 4 °C overnight with diluted primary antibody (mouse anti-ALP [1:1000, Abcam, Cat # ab126820], rabbit anti-RUNX2 (runt-related transcription factor 2) [1:1000, Cell Signaling Technology, Cat # 12556S], rabbit anti-beta-actin [1:1000, Cell Signaling Technology, Cat # 4970S], rabbit anti-talin1 [1:1000, Cell Signaling Technology, Cat #4021S], mouse anti-vinculin [1:1000, Sigma, Cat # V9131], mouse anti-FAK [1:500, Santa Cruz, Cat # sc1688], rabbit anti-YAP [1:1000, Cell Signaling Technology, Cat # 14074S], rabbit anti-GAPDH (glyceraldehyde-3-phosphate dehydrogenase) [1:5000, Bioworld, Cat # ap0063]), washed and incubated with horseradish peroxidase-conjugated secondary antibody (1:5000, Beyotime) at room temperature for 1 h. Enhanced chemiluminescence (ECL) chromogenic substrate was applied to enhance immunoreactive protein bands.

### Immunofluorescence analysis

Cells were fixed in 4% paraformaldehyde solution for 15 min at room temperature. Then, cells were cultured with 0.1% Triton X‐100 for 5 min at room temperature. Cells were subsequently incubated with 5% bovine serum albumin/phosphate‐buffered saline (PBS) for 30 min at room temperature. Cells were incubated with primary antibody overnight at 4 °C (mouse anti-integrin αVβ3 [1:200, Millipore, Cat # MAB1976], mouse anti-vinculin [1:200, Sigma], rabbit anti-YAP [1:200, Cell Signaling Technology], Phalloidin Red-594 [1:500, Beyotime, Cat # C2203S]). After washing with PBS, cells were incubated with secondary antibody for 1 h in the dark. A fluorescence microscope was used to view and acquire images.

### Statistical analysis

Statistical analysis was performed using SPSS 20.0 software (IBM, Inc. Armonk, NY, the USA). All data are presented as mean ± SD of three independent experiments. Data among three or more groups were analyzed using a one-way analysis of variance with Tukey’s post hoc analysis. Differences between two groups were analyzed using Student’s *t* test. A two-tailed value of *P* < 0.05 was considered statistically significant.

## Results

### Cyclic tensile stress promotes cell proliferation and osteogenesis in fibroblasts

**Fig. 1:**
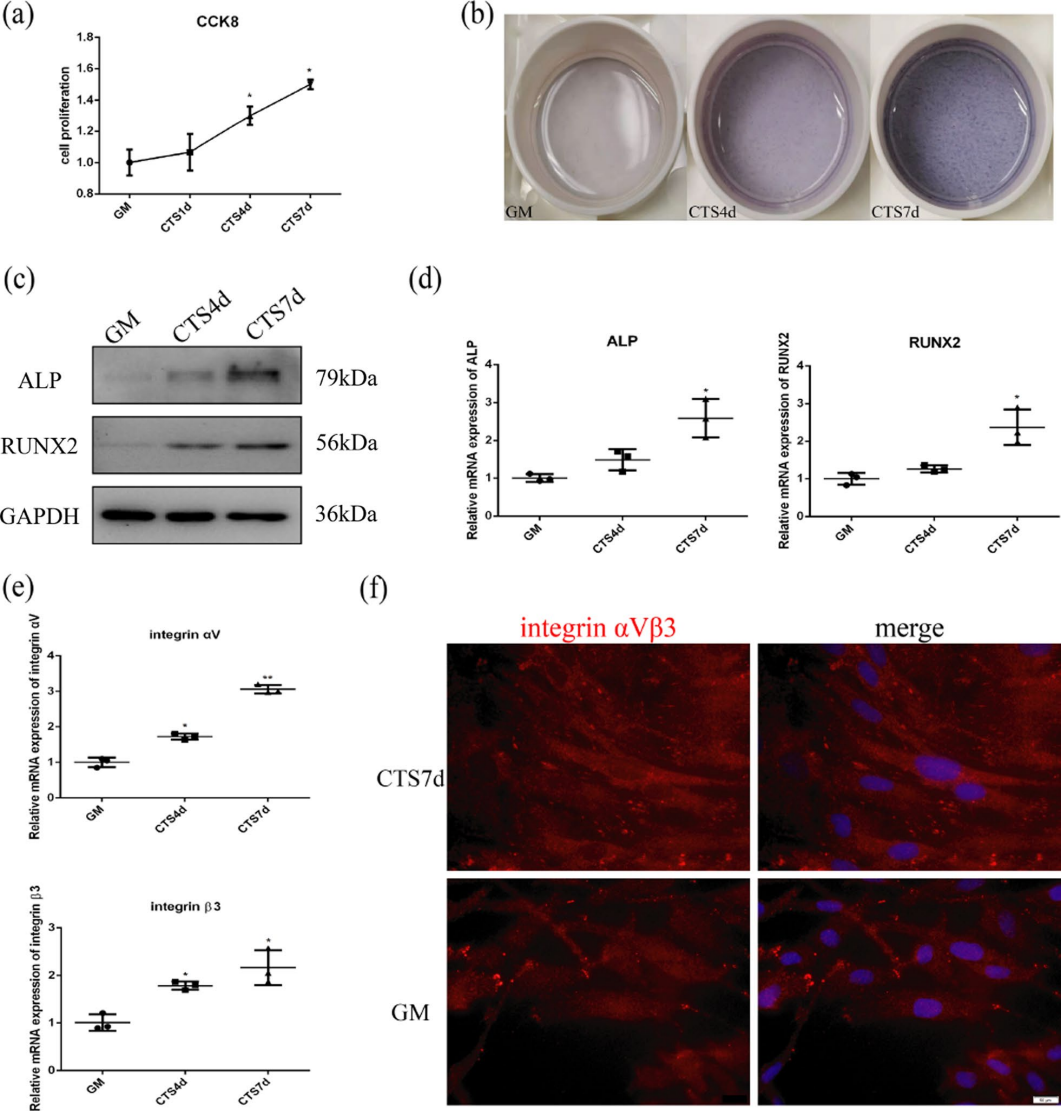
Cell proliferation, early stage of osteogenic differentiation and expression of integrin αVβ3 in fibroblasts under CTS. **a** Cell proliferation under CTS for 1 d, 4 d, 7 d. **b** ALP staining under CTS for 4 d and 7 d. **c** Protein expression of ALP and RUNX2 under CTS for 4 d and 7 d. **d** mRNA expression of ALP and RUNX2 under CTS for 4 d and 7 d. **e** mRNA expression of integrin αVβ3 under CTS for 4 d and 7 d. **f** Immunofluorescence of integrin αVβ3 under CTS for 7 d. Scale bar, 50 μm

This study explored the association between cyclic tensile stress, cell proliferation, and osteogenic differentiation of fibroblasts. First, fibroblasts were exposed to cyclic tensile stress for 4 d and 7 d. As shown in Fig. 1a, fibroblast proliferation increased in response to cyclic tensile stress. Protein and RNA expression of ALP and RUNX2, as markers of osteogenic differentiation, increased after 7 d under cyclic tensile stress (Fig. 1c, d). Similar results were obtained by ALP staining of fibroblasts, which was higher under cyclic tensile stress (Fig. 1b). These results suggest that cyclic tensile stress promotes cell proliferation and early stage of osteogenic differentiation in fibroblasts.

### Early stage of osteogenic differentiation induced by cyclic tensile stress depends on expression and activation of integrin αVβ3

**Fig. 2:**
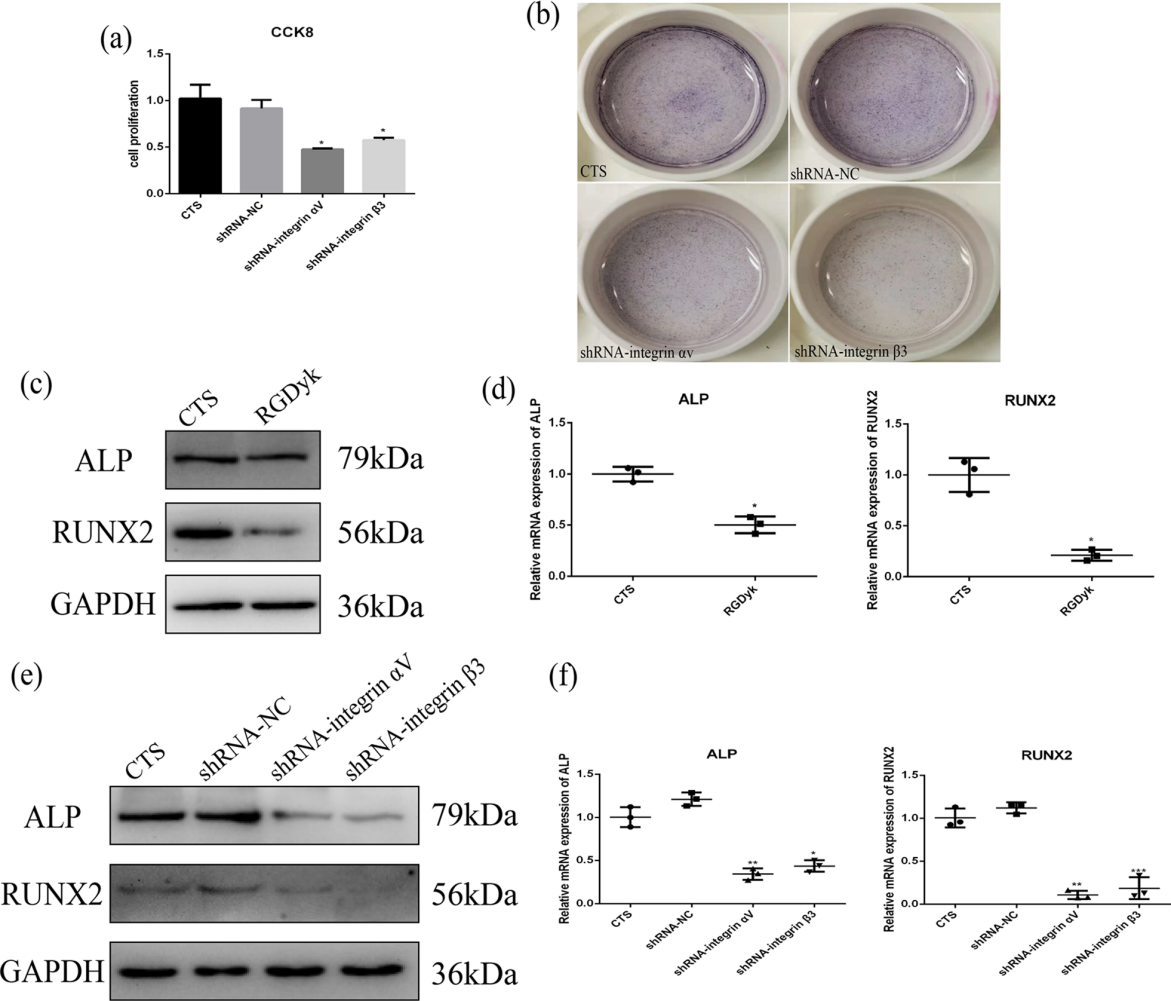
CTS regulated cell proliferation and early stage of osteogenic differentiation of fibroblasts via integrin αVβ3. **a** Cell proliferation under CTS in the GM, NC, shRNA-integrin αV and shRNA-integrin β3 groups. **b** ALP staining under CTS in the GM, NC, shRNA-integrin αV and shRNA-integrin β3 groups. **c** Protein expression of ALP and RUNX2 under CTS in the GM and RGDyk groups. **d** mRNA expression of ALP and RUNX2 under CTS in the GM and RGDyk groups. **e** Protein expression of ALP and RUNX2 under CTS in the GM, NC, shRNA-integrin αV and shRNA-integrin β3 groups. **f** mRNA expression of ALP and RUNX2 under CTS in the GM, NC, shRNA-integrin αV and shRNA-integrin β3 groups

To explore whether integrins are involved in osteogenic differentiation and cell proliferation induced by cyclic tensile stress, expression of integrin αVβ3 was analyzed in fibroblasts by qRT-PCR and immunofluorescence. We found that integrin αVβ3 expression increased under cyclic tensile stress (Fig. 1e, f), suggesting that cyclic tensile stress promotes expression of integrin αVβ3. Therefore, we investigated the association between integrin αVβ3 and osteogenic differentiation under cyclic tensile stress. To determine whether cyclic tensile stress stimulates osteogenic differentiation in an integrin αVβ3-dependent manner, the function of integrin αVβ3 was inhibited by shRNA or RGDyk, antagonists of integrin αVβ3 (Additional file 1: Fig. S1). As shown in Fig. 2a, when expression of integrin αV or β3 was downregulated in fibroblasts, cell proliferation decreased under cyclic tensile stress. Then, we detected the effect of integrin αVβ3 on osteogenic differentiation by ALP staining, western blotting and qRT-PCR. ALP staining was less in the shRNA-integrin αV and β3 groups than in the CTS group (Fig. 2b). Western blots showed that protein expression of ALP and RUNX2 decreased upon downregulation of integrin αVβ3 expression in fibroblasts (Fig. 2c, e). Similar results were obtained by qRT-PCR, with expression of ALP and RUNX2 mRNA decreasing when integrin αVβ3 expression was inhibited (Fig. 2d, f). Overall, these results show that cyclic tensile stress promotes early stage of osteogenic differentiation and cell proliferation of fibroblasts via integrin αVβ3.

### Cyclic tensile stress-regulated actin filaments via integrin αVβ3 during osteogenesis

**Fig. 3:**
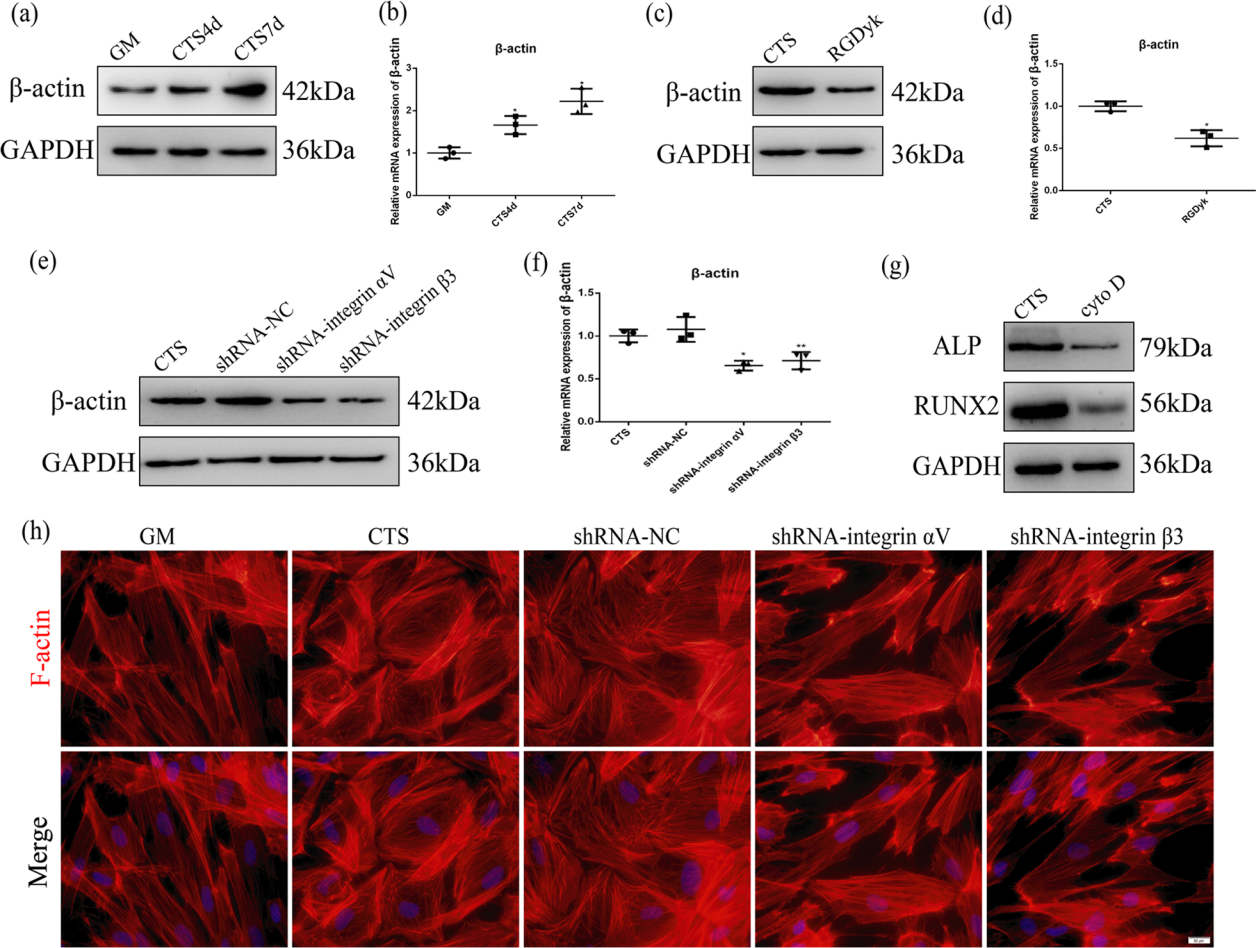
Actin filaments regulated early stage of osteogenic differentiation induced by CTS via integrin αVβ3 activation in fibroblasts. **a** Protein expression of β-actin under CTS for 4 d and 7 d. **b** mRNA expression of β-actin under CTS for 4 d and 7 d. **c** Protein expression of β-actin under CTS in the GM and RGDyk groups. **d** mRNA expression of β-actin under CTS in the GM and RGDyk groups. **e** Protein expression of β-actin under CTS in the GM, NC, shRNA-integrin αV and shRNA-integrin β3 groups. **f** mRNA expression of β-actin under CTS in the GM, NC, shRNA-integrin αV and shRNA-integrin β3 groups. **g** Protein expression of β-actin under CTS in the CTS and Cyto D groups. **h** Immunofluorescence of F-actin under CTS in the GM, CTS, NC, shRNA-integrin αV and shRNA-integrin β3 groups for 7 d. Scale bar, 50 μm

To better understand the association between integrin αVβ3 and actin filaments under cyclic tensile stress, we detected the expression of β-actin when integrin αVβ3 was inhibited. Western blotting and qRT-PCR showed that expression of β-actin increased under cyclic tensile stress (Fig. 3a, b). When expression of integrin αVβ3 was inhibited, expression of β-actin in the RGDyk group was lower than that in the CTS group (Fig. 3c, d). We also found that expression of β-actin in the shRNA-integrin αV or β3 groups was lower than that in the CTS group (Fig. 3e, f). Immunofluorescence showed that microfilament stress fibers were dispersed in a network structure close to the cell membrane under cyclic tensile stress. When expression of integrin αVβ3 was inhibited, microfilament stress fibers were arranged in an elongated manner similar to those in the GM group (Fig. 3h). These results suggest that cyclic tensile stress upregulates expression of β-actin and transforms the structure of actin filaments via integrin αVβ3.

To investigate whether actin filaments participate in osteogenic differentiation induced by cyclic tensile stress, we used cytochalasin D to inhibit polymerization of actin filaments [29, 30] (Figure S2a). Western blotting showed that expression of ALP and RUNX2 reduced by cytochalasin D under cyclic tensile stress (Fig. 3g). In summary, these results suggest that cyclic tensile stress regulates early stage of osteogenic differentiation of fibroblasts via the integrin αVβ3-microfilament axis.

### Cyclic tensile stress-regulated nuclear YAP via the integrin αVβ3-microfilament axis during osteogenesis

**Fig. 4:**
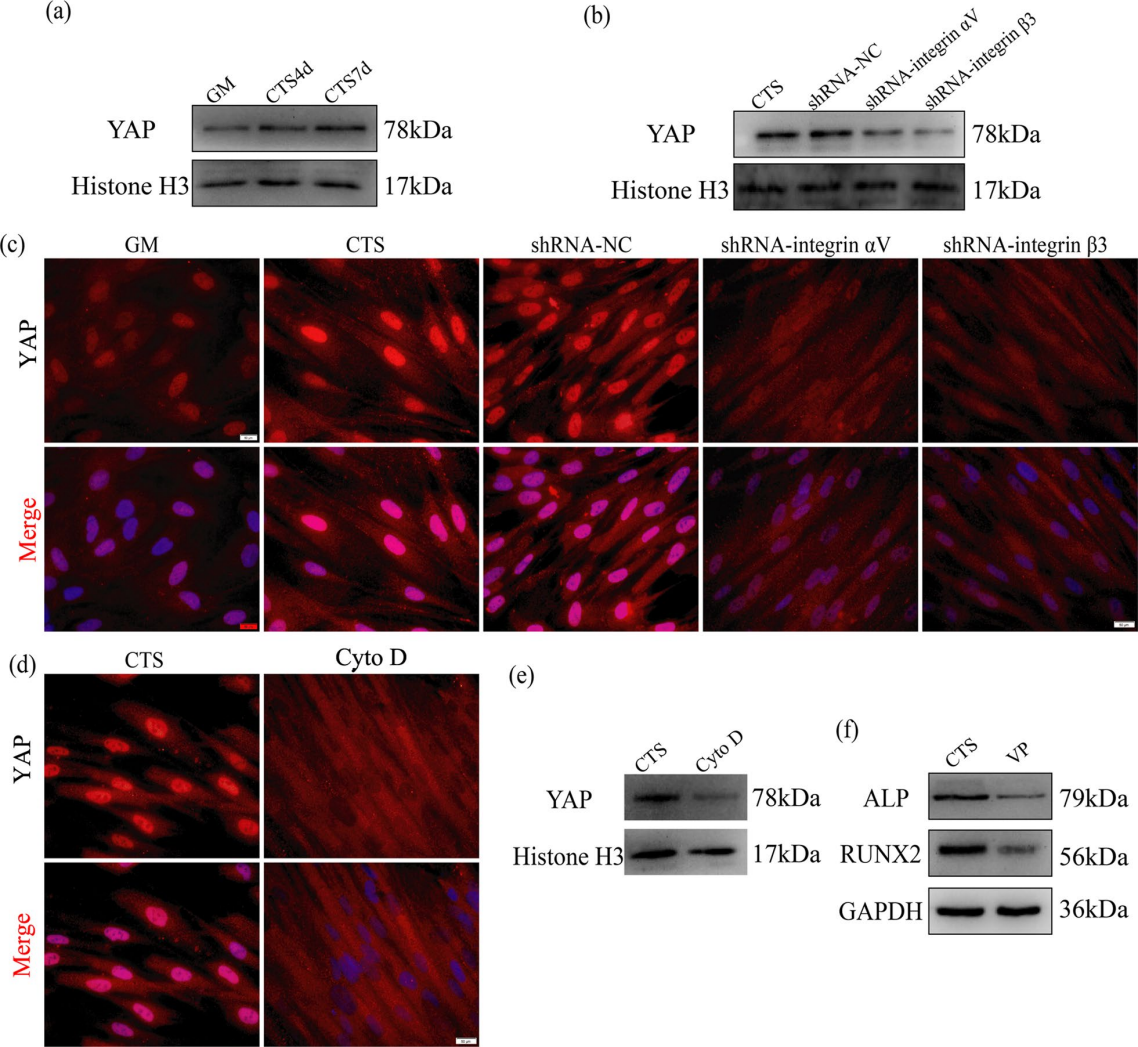
Integrin αVβ3 and actin filaments regulate expression of nuclear YAP during early stage of osteogenic differentiation induced by CTS. **a** Protein expression of nuclear YAP under CTS for 4 d and 7 d. **b** Protein expression of nuclear YAP under CTS in the GM, NC, shRNA-integrin αV and shRNA-integrin β3 groups. **c** Immunofluorescence of YAP under CTS in the GM, CTS, NC, shRNA-integrin αV and shRNA-integrin β3 groups for 7 d. Scale bar, 50 μm. **d** Immunofluorescence of YAP under CTS in the CTS and Cyto D groups for 7 d. Scale bar, 50 μm. **e** Protein expression of nuclear YAP under CTS in the CTS and Cyto D groups. **f** Protein expression of ALP and RUNX2 under CTS in the CTS and VP groups

We also explored whether cyclic tensile stress regulates expression of nuclear YAP via the integrin αVβ3-microfilament axis. Western blotting and qRT-PCR showed that expression of nuclear YAP increased under cyclic tensile stress (Fig. 4a). When expression of integrin αVβ3 was inhibited, western blotting showed that expression of nuclear YAP was lower than in the CTS group (Fig. 4b). The results of immunofluorescence showed that expression of nuclear YAP significantly increased under cyclic tensile stress. We also found that expression of nuclear YAP was reduced when integrin αVβ3 was inhibited (Fig. 4c). These results showed that cyclic tensile stress upregulated nuclear YAP via integrin αVβ3. When polymerization of β-actin was inhibited by cytochalasin D, western blotting and immunofluorescence showed that expression of nuclear YAP decreased under cyclic tensile stress (Fig. 4d, e). Western blotting also showed that expression of ALP and RUNX2 was reduced under cyclic tensile stress in the presence of verteporfin (Fig. 4f, Additional file 2: Fig. S2b). The above experiments show that cyclic tensile stress regulates nuclear YAP via the integrin αVβ3-microfilament axis during early stage of osteogenic differentiation.

### Cyclic tensile stress promoted the formation of focal adhesions and FAK phosphorylation during osteogenesis

**Fig. 5:**
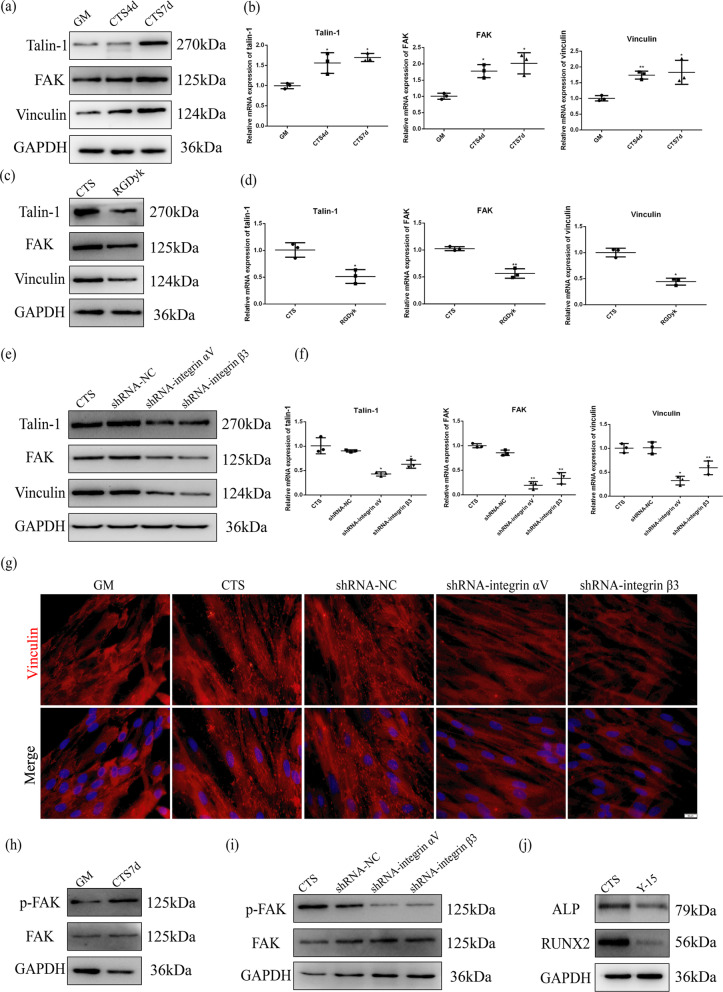
Integrin αVβ3 activation induced by CTS regulates focal adhesion-related proteins and phosphorylation of FAK to promote early stage of osteogenic differentiation. **a** Protein expression of talin-1, FAK and vinculin under CTS for 4 d and 7 d. **b** mRNA expression of talin-1, FAK and vinculin under CTS for 4 d and 7 d. **c** Protein expression of talin-1, FAK and vinculin under CTS in the GM and RGDyk groups. **d** mRNA expression of talin-1, FAK and vinculin under CTS in the GM and RGDyk groups. **e** Protein expression of talin-1, FAK and vinculin under CTS in the GM, NC, shRNA-integrin αV and shRNA-integrin β3 groups. **f** mRNA expression of talin-1, FAK and vinculin under CTS in the GM, NC, shRNA-integrin αV and shRNA-integrin β3 groups. **g** Immunofluorescence of vinculin under CTS in the GM, CTS, NC, shRNA-integrin αV and shRNA-integrin β3 groups for 7 d. Scale bar, 50 μm. **h** Phosphorylation level of FAK under CTS for 7 d. **i** Phosphorylation levels of FAK in the GM, NC, shRNA-integrin αV and shRNA-integrin β3 groups under CTS. **j** Protein expression of ALP and RUNX2 in the CTS and Y15 groups under CTS

Focal adhesions formed by actin filaments and integrins require talin, FAK and vinculin to transmit CTS to the substrate. We detected expression of talin-1, FAK and vinculin to explore their association with integrin αVβ3. Western blotting and qRT-PCR showed that expression of talin-1, FAK and vinculin increased under cyclic tensile stress (Fig. 5a, b). Western blotting showed that expression of talin-1, FAK and vinculin decreased when expression of integrin αVβ3 was inhibited by c(RGDyk) or shRNA (Fig. 5c, e). Similar results were obtained using qRT-PCR (Fig. 5d, f). Immunofluorescence showed that vinculin was more highly expressed and localized in visible focal adhesion plaques under cyclic tensile stress. Inhibition of integrin αVβ3 was associated with a significant reduction in vinculin expression (Fig. 5g). These results suggest that cyclic tensile stress promotes formation of focal adhesions via integrin αVβ3.

Next, we investigated the role phosphorylation of FAK plays in osteogenic differentiation under cyclic tensile stress. Western blotting showed that phosphorylation of FAK increased in response to cyclic tensile stress, and inhibition of integrin αVβ3 downregulated phosphorylation of FAK (Fig. 5h, i). We found that when phosphorylation of FAK was inhibited by Y-15, a small molecule inhibitor, expression of ALP and RUNX2 was reduced (Fig. 5j, Additional file 2: Fig. S2c, Fig. S3). These results show that not only does FAK participate in the formation of focal adhesions containing integrin αVβ3 but also that phosphorylation of FAK contributes to early stage of osteogenic differentiation induced by cyclic tensile stress.

## Discussion

Mechanotransduction is an important mechanism for regulating osteogenic differentiation of stem cells. Among various signaling pathways involved in osteogenic differentiation of stem cells, the ECM (extracellular matrix)-integrin-microfilament axis makes the greatest contribution to the response of stem cells to mechanical loading [31]. The present study showed that integrin αVβ3 contributed to early stage of osteogenic differentiation induced by cyclic tensile stress in fibroblasts.

Tensile stress has been shown to be an osteogenic stimulus for stem cells. In previous studies of the effects of tensile stress, stem cells were seeded onto a flexible membrane or within a matrix to which stress was applied [32–34]. Our study showed that cyclic tensile stress (10% strain, 0.5 Hz, 2 h/d) promotes cell proliferation and early stage of osteogenic differentiation of fibroblasts even without osteoblast-inducing conditioned media (Fig. 1a, b and Additional file 3: Fig. S3). However, differentiation of stem cells is dependent on characteristics of the applied tensile stress, including stress magnitude, frequency, and application time [35–38]. The relationship between tensile stress and osteogenic differentiation needs to be further explored. Research has shown that tensile stress can directly induce osteogenic differentiation of mesenchymal stem cells, but the specific targets of the tensile stress have yet to be fully characterized [39, 40]. Studies have shown that integrin αVβ3 mediates expression of sclerostin by periostin and hence osteogenesis and survival in response to mechanical stimulation [41]. Therefore, we considered whether integrin αVβ3 plays a role in early stage of osteogenic differentiation induced by cyclic tensile stress.

Integrins connect to the intracellular actin cytoskeleton and provide a mechanical foundation for conduction of forces, cell shape stabilization, cell migration and differentiation [42]. Studies have shown that activation of integrin αVβ3 by mechanical loading upregulates the expression of c-fos, IGF-1 and COX-2, which can affect calcium influx in osteoblasts [43]. Activation of integrins from a low-affinity state to a high-affinity state is the basis of the formation of focal adhesions. Activation of integrins also recruits and activates adaptor proteins to form visible focal adhesion complexes [44]. The combination of integrins and ECM ligands activates multiple signaling pathways, including phosphorylation of enzymes, calcium influx and activation of the Rho pathway, which influence various aspects of cell physiological activity [42]. FAK is an indispensable part of focal adhesion structure, and the co-localization of FAK and integrins in focal adhesions is a prerequisite of FAK phosphorylation [45, 46]. It is likely that there are several signaling pathways responsible for mechanical signal transmission between cells and the ECM that regulate stem cell differentiation. It has been shown that FAK may be an important signaling protein that reacts to mechanical loading in complex signaling networks [47, 48]. Our results show that integrin αVβ3 recruits talin, FAK and vinculin to form focal adhesions that promote osteogenic differentiation induced by cyclic tensile stress. Conversely, it has been reported that integrin αVβ3 affects osteoclast reabsorption by regulating cell polarity and cytoskeletal recombination [49]. Based on these results, we suggest that integrin αVβ3 plays an important role in bone physiology and the response to mechanical loading of osteocytes.

The microfilament cytoskeleton formed by actin polymerization plays an important role in osteogenic differentiation of mesenchymal stem cells [50]. The arrangement of the microfilament cytoskeleton is significantly altered during osteogenic differentiation. When stem cells differentiate into osteoblasts, the microfilament cytoskeleton becomes more dispersed as cells transform into osteoblasts [51]. Studies have shown that destruction of the microfilament cytoskeleton downregulates osteogenic differentiation induced by flow shear stress [52]. Our study shows that rearrangement of the microfilament cytoskeleton contributes to early stage of osteogenic differentiation induced by cyclic tensile stress. In addition, the regulation of YAP by mechanical loading requires the microfilament cytoskeleton. Studies have shown that expression of nuclear YAP can be stimulated by actin polymerization [53–55]. The results of this study suggest that expression of nuclear YAP can be regulated by activation of the integrin αVβ3-microfilament axis under cyclic tensile stress. In addition, a recent study showed that YAP promotes osteogenic differentiation by regulating β-catenin signaling [56]. Therefore, we suggest that YAP plays different roles in multiple signaling pathways related to early stage of osteogenic differentiation.

## Conclusion

Cyclic tensile stress exerts a positive effect on early stage of osteogenic differentiation of fibroblasts via integrin αVβ3 activation, leading to rearrangement of the microfilament cytoskeleton. Furthermore, rearrangement of actin filaments and phosphorylation of FAK stimulated by cyclic tensile stress via integrin αVβ3 lead to increased expression of nuclear YAP in human fibroblasts. In future studies, integrin αVβ3 should be considered a promising target for in vivo experiments to verify its function in the promotion of bone repair.

## Supplementary Information

**Additional file 1: Fig. S1**. The function of integrin αVβ3 was inhibited by c(RGDyk) (10 μM) (a) and Lentivirus transduction (b, c).

**Additional file 2: Fig. S2**. (a) Polymerization of β-actin was inhibited by cytochalasin D (0.2 μg/mL). (b) The function of nuclear YAP was inhibited by verteporfin (5 μM). (c) Phosphorylation of FAK was inhibited by Y-15 (2 μM).

**Additional file 3: Fig. S3**. Adipogenic differentiation and Chondrogenic differentiation in fibroblasts under CTS. (a) Oil-red O staining under CTS for 7 d. (b) Toluidine blue staining under CTS for 7 d.

**Additional file 4: Fig. S4**. Original images of western blots.

## Data Availability

All the supporting data can be downloaded.

## Abbreviations

ALP: Alkaline phosphatase
Cyto D: Cytochalasin D
CST: Cyclic tensile stress
ECM: Extracellular matrix
ECL: Enhanced chemiluminescence
FAK: Focal adhesion kinase
GAPDH: Glyceraldehyde-3-phosphate dehydrogenase
GM: Growth medium
NC: Negative control
PBS: Phosphate-buffered saline
PVDF: Polyvinylidene difluoride
RUNX2: Runt-related transcription factor 2
TEAD: TEA domain DNA-binding family of transcription factors
VP: Verteporfin
YAP: Yes-associated protein
