# Supplementary figures and images for: Regulation of the integrin αVβ3- actin filaments axis in early osteogenesis of human fibroblasts under cyclic tensile stress
Source: Stem Cell Res Ther. 2021 Oct 7;12:523. doi: 10.1186/s13287-021-02597-y (PMC8496073; doi:10.1186/s13287-021-02597-y)

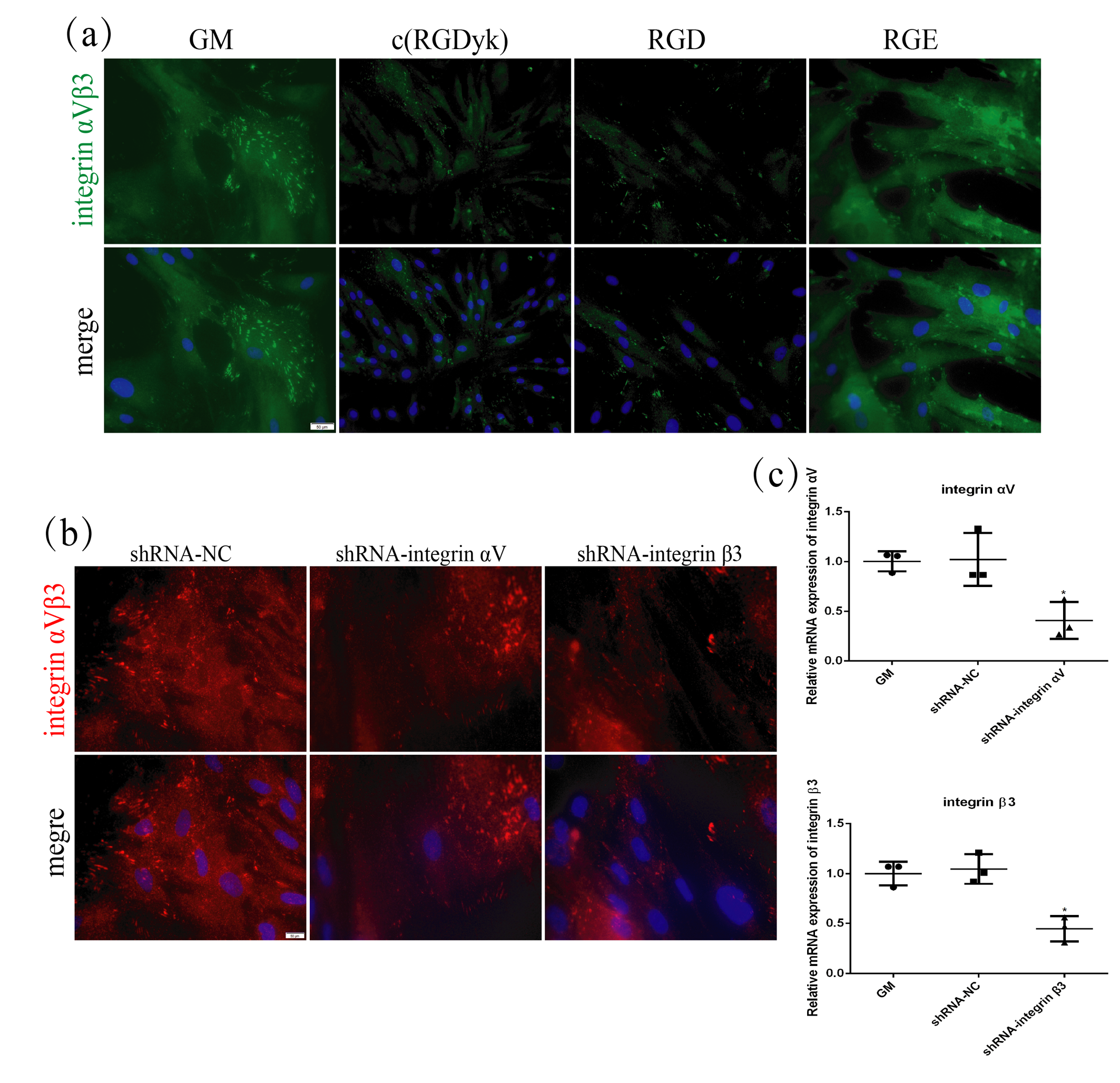

Supplement: Supplementary file 1 — Additional file 1: Fig. S1. The function of integrin αVβ3 was inhibited by c(RGDyk) (10 μM) (a) and Lentivirus transduction (b, c). [file 13287_2021_2597_MOESM1_ESM.tif]

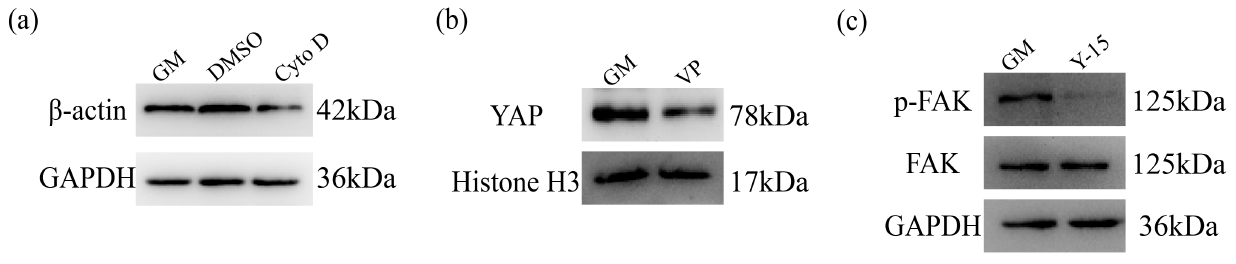

Supplement: Supplementary file 2 — Additional file 2: Fig. S2. (a) Polymerization of β-actin was inhibited by cytochalasin D (0.2 μg/mL). (b) The function of nuclear YAP was inhibited by verteporfin (5 μM). (c) Phosphorylation of FAK was inhibited by Y-15 (2 μM). [file 13287_2021_2597_MOESM2_ESM.tif]

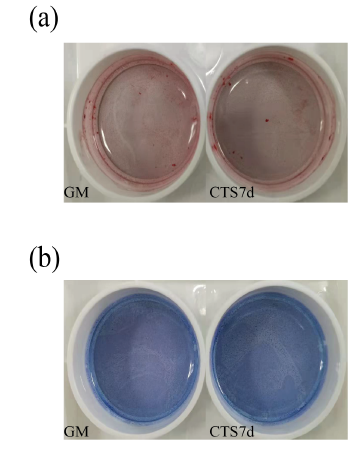

Supplement: Supplementary file 3 — Additional file 3: Fig. S3. Adipogenic differentiation and Chondrogenic differentiation in fibroblasts under CTS. (a) Oil-red O staining under CTS for 7 d. (b) Toluidine blue staining under CTS for 7 d. [file 13287_2021_2597_MOESM3_ESM.tif]

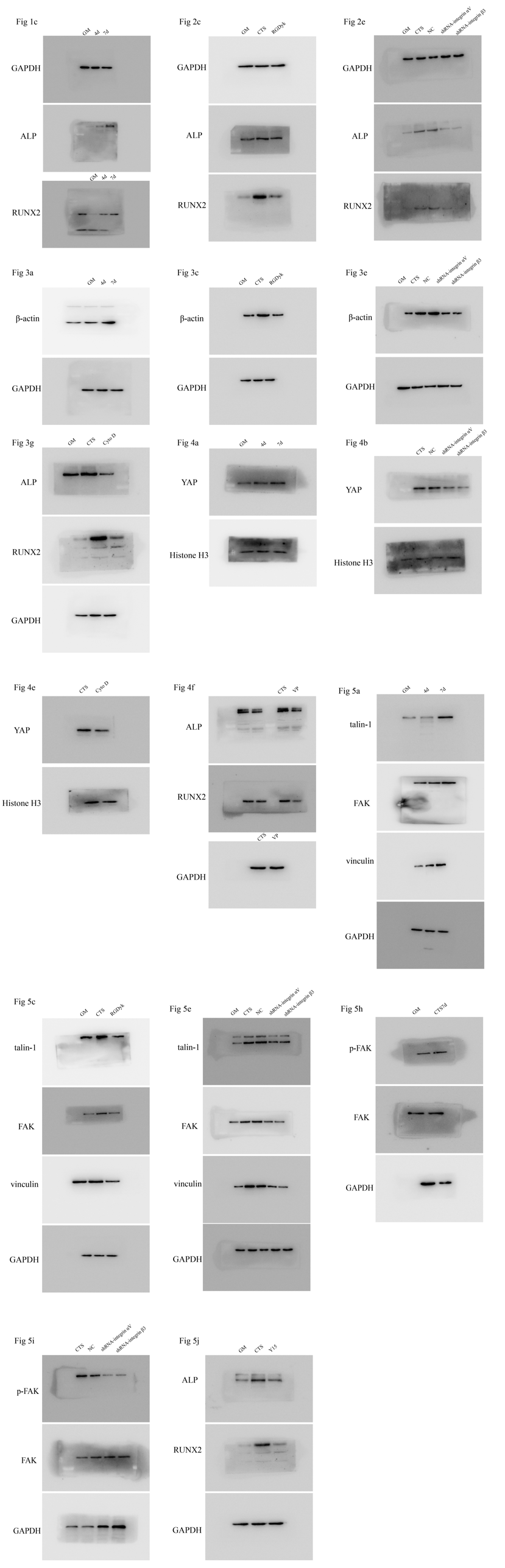

Supplement: Supplementary file 4 — Additional file 4: Fig. S4. Original images of western blots. [file 13287_2021_2597_MOESM4_ESM.tif]
